# Effects of Jasmonic Acid in ER Stress and Unfolded Protein Response in Tomato Plants

**DOI:** 10.3390/biom10071031

**Published:** 2020-07-10

**Authors:** Zalán Czékus, Orsolya Csíkos, Attila Ördög, Irma Tari, Péter Poór

**Affiliations:** 1 Department of Plant Biology, University of Szeged, Közép fasor 52, H-6726 Szeged, Hungary; czekus.z@bio.u-szeged.hu (Z.C.); borsika94@hotmail.com (O.C.); aordog@bio.u-szeged.hu (A.Ö.); tari@bio.u-szeged.hu (I.T.); 2 Doctoral School of Biology, University of Szeged, Közép fasor 52, H-6726 Szeged, Hungary

**Keywords:** ER stress, jasmonic acid, nitric oxide, protein carbonylation, reactive oxygen species, unfolded protein response

## Abstract

Endoplasmic reticulum (ER) stress elicits a protective mechanism called unfolded protein response (UPR) to maintain cellular homeostasis, which can be regulated by defence hormones. In this study, the physiological role of jasmonic acid (JA) in ER stress and UPR signalling has been investigated in intact leaves of tomato plants. Exogenous JA treatments not only induced the transcript accumulation of UPR marker gene *SlBiP* but also elevated transcript levels of *SlIRE1* and *SlbZIP60*. By the application of JA signalling mutant *jai1* plants, the role of JA in ER stress sensing and signalling was further investigated. Treatment with tunicamycin (Tm), the inhibitor of N-glycosylation of secreted glycoproteins, increased the transcript levels of *SlBiP*. Interestingly, *SlIRE1a* and *SlIRE1b* were significantly lower in *jai1*. In contrast, the transcript accumulation of *Bax Inhibitor-1* (*SlBI1*) and *SlbZIP60* was higher in *jai1*. To evaluate how a chemical chaperone modulates Tm-induced ER stress, plants were treated with sodium 4-phenylbutyrate, which also decreased the Tm-induced increase in *SlBiP*, *SlIRE1a,* and *SlBI1* transcripts. In addition, it was found that changes in hydrogen peroxide content, proteasomal activity, and lipid peroxidation induced by Tm is regulated by JA, while nitric oxide was not involved in ER stress and UPR signalling in leaves of tomato.

## 1. Introduction

A wide range of biotic and abiotic stress factors can disrupt the protein-folding capacity and the transport balance of the endoplasmic reticulum (ER), which results in the accumulation of misfolded or unfolded proteins in the lumen of ER and thus induces ER stress in plants [1,2,3,4]. The disturbances in ER homeostasis trigger an evolutionarily conserved response, termed the unfolded protein response (UPR). UPR is a protective process to maintain the cellular homeostasis by regulating the expression of variety of genes (e.g., chaperones) and by reducing protein loading to ER and enhancing ER-associated protein degradation (ERAD) [4,5,6,7]. Programmed cell death (PCD) and autophagy are also in tight connection with ERAD response, especially under chronic stress [8,9,10,11]. These ER-mediated stress responses in plants can be regulated by phytohormones but the activation of the UPR pathway by defence-related hormones remains mostly unclear [12].

At least two UPR pathways have been identified in plants. One of them, the inositol-requiring enzyme 1 (IRE1)-mediated unconventional splicing of basic leucine zipper (bZIP) 60 is the most characterised pathway in eukaryotes [13,14,15]. Briefly, the accumulation of unfolded proteins leads to BiP (luminal binding protein) dissociation from IRE1, which becomes firstly dimerized, then finally oligomerized in the ER membrane. Following that, the activated RNAse function of IRE1 results in the splicing of bZIP60 mRNA and the bulk degradation of selected mRNAs through regulated IRE1-dependent decay (RIDD) in yeast, animals, and plants. The spliced bZIP60 mRNA is translated to an active transcription factor (TF) and by up-regulating the UPR-related genes helps the protein folding and degradation [13,16,17,18,19]. The activation of ER membrane-anchored TF bZIP28 and the plant B-cell lymphoma2 (Bcl-2)-associated athanogene 7 (BAG7) protein is another mode to control ER stress in plants. Based on the investigation in *Arabidopsis*, both proteins are anchored to the ER membrane by interactions with BiP, which is an ER chaperone under control condition. bZIP28 is also activated through the stress-induced accumulation of unfolded proteins in the ER lumen. BiP dissociates from bZIP28 and the released bZIP28 translocates from ER to the Golgi, where it is proteolytically cleaved by Site-2 protease (S2P) under ER stress. The cleaved form of bZIP28 moves into the nucleus and binds to the ER stress element (ERSE) *cis*-regulatory motifs to activate the UPR gene expression [19,20,21,22,23,24]. BAG7 is also released from the ER membrane by an unknown protease; then, it is sumoylated and enters the nucleus, where it interacts with WRKY29 transcription factor and regulates the expression of various chaperone proteins to mitigate ER stress in *Arabidopsis* [25]. Another TF, the plant-specific NAM, ATAF, and CUC (NACs) TFs (e.g., NAC062 and NAC089) have been recently described as important regulators of ER stress responses that also undergo proteolytic cleavage and translocation to the nucleus to promote the transcription of UPR or PCD genes in *Arabidopsis* [2,4]. Unfortunately, most of the components of ER stress and UPR are identified in the *Arabidopsis* model plant, and only a few studies have examined these processes in crops, where the knowledge of the homologue signalling components is very limited [12]. The investigation of ER stress and UPR in cultivated plants could have great importance under diverse biotic and abiotic stress conditions from the agricultural aspect.

Since UPR plays a fundamental role in plant immunity and stress responses, the potential regulatory role of defence hormones in this process is relevant under the changing environment [12]. It has been demonstrated that various environmental stresses such as salinity or high temperature can cause ER stress and induce UPR in plants [26,27,28]. In conjugation with these, it has been demonstrated that several phytohormones are involved in the induction of ER stress and regulation of UPR, such as auxin or salicylic acid (SA) [29,30,31]. Although the role of SA in ER stress signalling and UPR has been almost fully characterised recently, the underlying mechanisms, interactions, and the potential molecular and physiological role of other defence-related phytohormones such as jasmonic acid (JA) has not yet been completely described [4,12,32,33]. Besides SA, JA also plays a crucial role during the plant–pathogen interactions and in resistance processes upon abiotic stressors [34,35,36,37,38,39]. JA promotes the production of antifungal proteins, such as defensins and protease inhibitors within hours after the infection or wounding, as well as increases the activity of various defence enzymes [37,40]. The rapid and optimal coordination of protein secretory machinery is required to ensure folding, modification, and transport of stress-related proteins, but the potential role of JA in these processes and UPR is not known in full detail. Interaction between JA and UPR marker BiP chaperone was confirmed recently in BiP-overexpressing transgenic soybean plants, where JA content was significantly lower compared to wild-type (WT) plants under control condition [41]. Firstly, Moreno et al. [42] tested the effects of methyl jasmonate (MeJA) in *Arabidopsis*. Their results showed that 0.03 mM MeJA failed to activate bZIP60 mRNA processing within 5 h. More recently, Xu et al. [33] demonstrated that MeJA in the concentration of 1 mM can transiently induce UPR marker genes within 3 h. The expression of *BiP*, *protein disulfide isomerase* (*PDI*), *calnexin* (*CNX*), and *calreticulin* (*CRT*) was at its maximum after 3 h but the transcript levels of *bZIP60* were significantly higher after 12 h in *Arabidopsis* exposed to 1 mM MeJA [33]. These gene expression data suggest a rapid and concentration-dependent effect of MeJA, but the biochemical and physiological changes have neither been investigated in *Arabidopsis* nor in crops. Among others, the production of reactive oxygen species (ROS), lipid and protein oxidation, and the regulated and non-regulated degradation of damaged proteins are key components of these processes [43], but the role and effects of JA are less known.

JA receptor and signalling mutants make it possible to analyse not only the molecular steps of signalling but also the physiological functions of JA under stress conditions. The *jasmonic acid–insensitive1* (*jai1*), which is defective in JA signalling, is one of the best-characterised tomato mutants. It was described earlier that these sterile mutants cannot express JA-responsive genes and cannot accumulate JA-regulated proteinase inhibitors. Moreover, these mutant plants are severely resistant to two-spotted spider mites [44]. To test the potential role of JA in UPR, exogenous treatment with tunicamycin (Tm, the inhibitor of N-glycosylation of secreted glycoproteins) can provide an opportunity to provoke ER stress in leaf tissues under laboratory conditions [14].

In this work, the rapid effect of JA was investigated in ER stress sensing and signalling in intact leaves of tomato plants. Changes in the transcript accumulation of UPR marker *BiP* gene, as well as the transcript levels of ER stress sensor *SlIRE1* and UPR transcription factor *SlbZIP60* were also detected upon exogenous JA treatments. The potential physiological role of JA in ER stress sensing and UPR was investigated the first time in *jai1* plants which are defective in JA signalling. Our experiments were focused on the JA-mediated ROS and nitric oxide (NO) production, as well as lipid and protein oxidation under UPR.

## 2. Materials and Methods

### 2.1. Plant Material and Growth Conditions

Seeds of wild-type (WT) and jasmonic acid–insensitive1 (jai1) tomato plants (*Solanum lycopersicum* L. cv. Castlemart) were germinated for 3 days at 26 °C under darkness (Seeds were kindly provided by Dr. Bettina Hause (Leibniz Institute of Plant Biochemistry, Halle, Germany) and Dr. Gregg Howe (Michigan State University, East Lansing, MI, USA)). Homozygous *jai-1/jai-1* seedlings were pre-selected by treatment with 1 mM methyl jasmonate (MeJA) during the germination [45]. MeJA-insensitive seedlings (*jai-1/jai-1*) did not suffer any growth inhibition or showed a violet colour compared to the sensitive ones exposed to MeJA. The selected seedlings were transferred to perlite for 14 days; then, they were placed in hydroponic culture and plants were grown under a controlled environment (200 μmol m^−2^ s^−1^ photon flux density, F36W/GRO lamps (OSRAM SYLVANIA, Danvers, MA, USA) with 12/12-h light/dark period, day/night temperatures of 24/22 °C and a relative humidity of 55–60%) [46]. Before the experiments, the insensitive plants were subjected to a PCR analysis to select the homozygous ones [44].

### 2.2. Chemical Treatments

Tomatoes at the five-leaf stage were treated by adding 1 μM or 10 μM JA to the nutrient solution for 6 h to test the effects of JA on WT plants. Both genotypes were subjected to the treatment of the ER stress-inducer Tm (0.5 μg mL^−1^) via the nutrient solution. To evaluate the effects of chemical chaperone on Tm-induced ER stress, plants were incubated in the nutrient solution with 1 mM sodium 4-phenylbutyrate (PBA) chemical chaperone in the presence of 0.5 μg mL^−1^ Tm for 6 h [47]. All chemicals were purchased from Sigma-Aldrich (Sigma-Aldrich, St. Louis, MO, USA). All experiments were started at 9:00 (3 h after light on) and were repeated three times. The samples were prepared from fully expanded leaves in three replicates, 6 h after the treatments.

### 2.3. Analyses of Transcript Accumuation by Quantitative Real-Time PCR

Total RNA was isolated from leaves using the TRI reagent method [48]. Genomic DNA digestions were performed by DNase I (Fermentas UAB, Vilnius, Lithuania) and the first-strand cDNA was synthesized using MMLV reverse transcriptase (Fermentas UAB, Vilnius, Lithuania). Quantitative real-time-PCR (qRT-PCR; qTOWER Real-Time qPCR System, Analytik Jena, Jena, Germany) was used to detect the transcript accumulation of the selected tomato genes (Table 1) mined from the National Center for Biotechnology Information Search database [49].

The PCR reaction mixture contained 10 ng cDNA template, 400–400 nM forward and reverse primers, 5 µL of Maxima SYBR Green qPCR Master Mix (2X) (Thermo Scientific, Waltham, MA, USA) and nuclease-free water in 10 μL volume (denaturation at 95 °C for 7 min, followed by 40 cycles of denaturation at 95 °C for 15 s and annealing extension at 60 °C for 60 s). qTOWER Software 2.2 (Analytik Jena, Jena, Germany) was used to analyse the data [50]. Tomato 18S rRNA and elongation factor-1α subunit were applied as reference genes, and the data were calculated by the 2^(−∆∆Ct)^ formula [51].

### 2.4. Determination of Hydrogen Peroxide (H_2_O_2_) Content

First, 200 mg of leaf tissue was homogenised with 1 mL of ice-cold, 0.1% trichloroacetic acid (TCA). Then, the samples were centrifuged (11,500× *g* for 10 min at 4 °C). Then, 0.25 mL of the supernatant was added to the reaction mixture, which contained 0.25 mL of 50 mM phosphate buffer (pH 7.0) and 0.5 mL of 1 M potassium iodide (KI). After 10 min, the absorbance of the samples was measured at 390 nm with spectrophotometer [52]. The amount of H_2_O_2_ was calculated using a standard curve of H_2_O_2_ solution (Sigma-Aldrich, St. Louis MO, USA).

### 2.5. Detection of Nitric Oxide (NO) Production

NO production in tomato leaves was visualized using 4-amino-5-methylamino-2′,7′-difluorofluorescein diacetate (DAF-FM DA) (Sigma-Aldrich, St. Louis, MO, USA). Leaf discs were infiltrated with 10 μM DAF-FM DA under vacuum for 30 min (the dye was solved in 10 mM Tris-HCl, pH 7.4) in the dark at room temperature. Following the infiltration, the samples were rinsed twice with 10 mM Tris-HCl (pH 7.4). Fluorescence intensity of stained leaf samples was detected with a Zeiss Axiowert 200M-type fluorescence microscope (Carl Zeiss Inc., Jena, Germany) equipped with a high-resolution digital camera (Axiocam HR, Carl Zeiss Inc., Jena, Germany). Data were analysed by AXIOVISION REL. 4.8 software (Carl Zeiss Inc., Munich, Germany) [53].

### 2.6. PROTEIN Extraction

Soluble proteins were extracted from 250 mg leaf tissues with 1 mL of 50 mM sodium-acetate buffer (pH 6.1) at 4 °C. The extract was centrifuged (11,500× *g*, 4 °C, 10 min); then, the total protein content of supernatants was determined by the method of Bradford et al. [54].

### 2.7. Determination of Protein Carbonylation

First, 750 μL of 10 mM 2,4-dinitrophenylhydrazine (DNPH) in 2 M HCl was added to 60 μg protein extract and incubated in the dark for 1 h being vigorously mixed every 10 min. The labelling was stopped by adding 0.5 mL of 20% TCA, and the samples were left on ice for 10 min. After centrifugation (11,500× *g*, 4 °C, 10 min), the pellet was washed three times with 1 mL of ethanol:ethyl acetate (1/1, *v*/*v*). After each washing step, the samples were centrifuged (10,000× *g*, 4 °C, 10 min). The final pellet was dissolved in 1 mL of 6 M guanidine hydrochloride (Sigma-Aldrich, St. Louis MO, USA) with 0.5 M potassium phosphate (pH 2.5) and incubated at room temperature for 10 min. After centrifugation (10,000× *g*, 4 °C, 10 min), the absorbance of the supernatant was measured at 375 nm with a spectrophotometer [55]. The concentration of carbonyls was calculated from the molar extinction coefficient for DNPH, ε375 = 22.000 M^−1^ cm^−1^.

### 2.8. Determination of Proteasomal Activity

First, 10 μL of protein extract was incubated at 37 °C for 60 min with 90 μL of 50 μM succinyl-Leu-Leu-Val-Tyr-7-amido-4-methylcoumarin (Suc-LLVY-AMC; Sigma-Aldrich, St. Louis MO, USA) dissolved in 80 mM potassium phosphate (pH 7.0) under darkness. To determine the specific proteasomal activity, 10 μM MG132 (Carbobenzoxy-l-leucyl-l-leucyl-l-leucinal; Sigma-Aldrich, St. Louis MO, USA) dissolved in DMSO was used in parallel during the incubation. The reaction was stopped by adding 0.3 mL of 80 mM sodium acetate (pH 4.3). Released AMC was monitored by fluorescence using FLUOstar Optima Microplate Reader (BMG Labtech, Ortenberg, Germany). The excitation and emission wavelengths were 380 and 440 nm, respectively. Data were plotted in relative fluorescence units (RFU) per protein content of each fraction [56].

### 2.9. Determination of Protease Activity

First, 50 μL of protein extract was incubated in 650 μL of potassium phosphate buffer (pH 5.5) and 0.3 mL of 1% azocasein (*w*/*v* in 0.1 N NaOH; Sigma-Aldrich, St. Louis MO, USA) for 2 h at 37 °C in dark. The reaction was stopped by adding 300 μL of 10% (*w*/*v*) TCA and left on ice for 20 min. Samples were centrifuged (15,500× *g*, 4 °C, 10 min); then, the yellow colour was measured at 440 nm with a spectrophotometer. One arbitrary unit (AU) of activity was defined as the amount of protein capable of increasing absorbance at 440 nm by 0.01 [57].

### 2.10. Determination of Malondialdehyde (MDA) Content

First, 100 mg of leaf samples were homogenised with 1 mL of 0.1% TCA. Before the centrifugation (11,500× *g*, 4 °C, 20 min) 0.1 mL of 4% butylated hydroxytoluene (BHT; Sigma-Aldrich, St. Louis MO, USA) was added to the samples. Then, 0.5 mL of supernatant was mixed with 2 mL 0.5% thiobarbituric acid (TBA; Sigma-Aldrich, St. Louis MO, USA) dissolved in 20% TCA, and the mixture was incubated in boiling water for 30 min. The absorbance was measured at 532 nm and adjusted for non-specific absorbance at 600 nm. Malondialdehyde (MDA) content was calculated using the extinction coefficient of 155 mM^−1^ cm^−1^ [58].

### 2.11. Determination the Electrolyte Leakage (EL)

First, 0.1 g of leaf discs were incubated in 20 mL of double-distilled water at 25 °C for 2 h under darkness. The conductivity in the bathing solution was first determined (C1) with a conductivity meter (HANNA Instruments, Woonsocket, RI, USA); then, the samples were heated at 95 °C for 40 min and the total conductivity (C2) of the cooled samples was also measured. Relative electrolyte leakage (EL) was expressed as EL (%) = (C1/C2) × 100 [59].

## 3. Results

**Figure 1:**
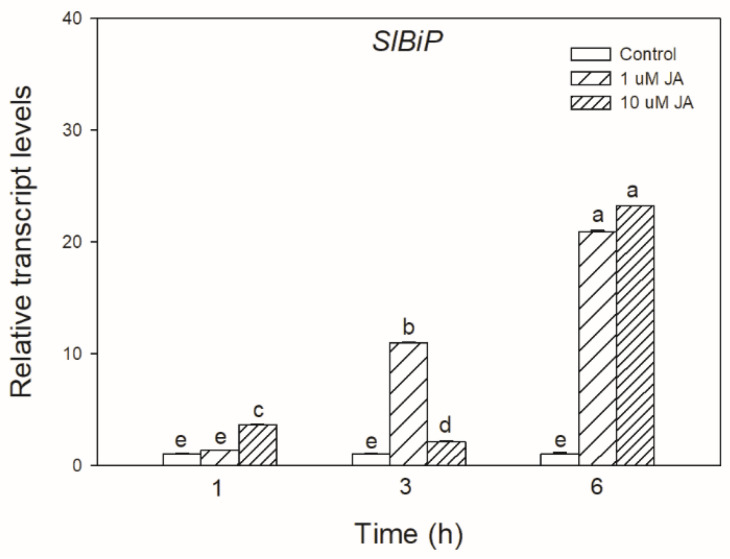
Changes in the transcript accumulation of *SlBiP* in the leaves of wild-type tomato plants exposed to 1 or 10 µM jasmonic acid (JA) for 6 h. Results are the means ± SE. Data with different letters indicate significant differences at *p* < 0.05 level, *n* = 3.

To test the possible effect of JA in UPR signalling, the transcript accumulation of *SlBiP* chaperon-coding gene was firstly examined after exogenous JA treatments in tomato leaves. It was found that both applied JA concentrations drastically elevated the transcript levels of *SlBiP* after 6 h (Figure 1).

**Figure 2:**
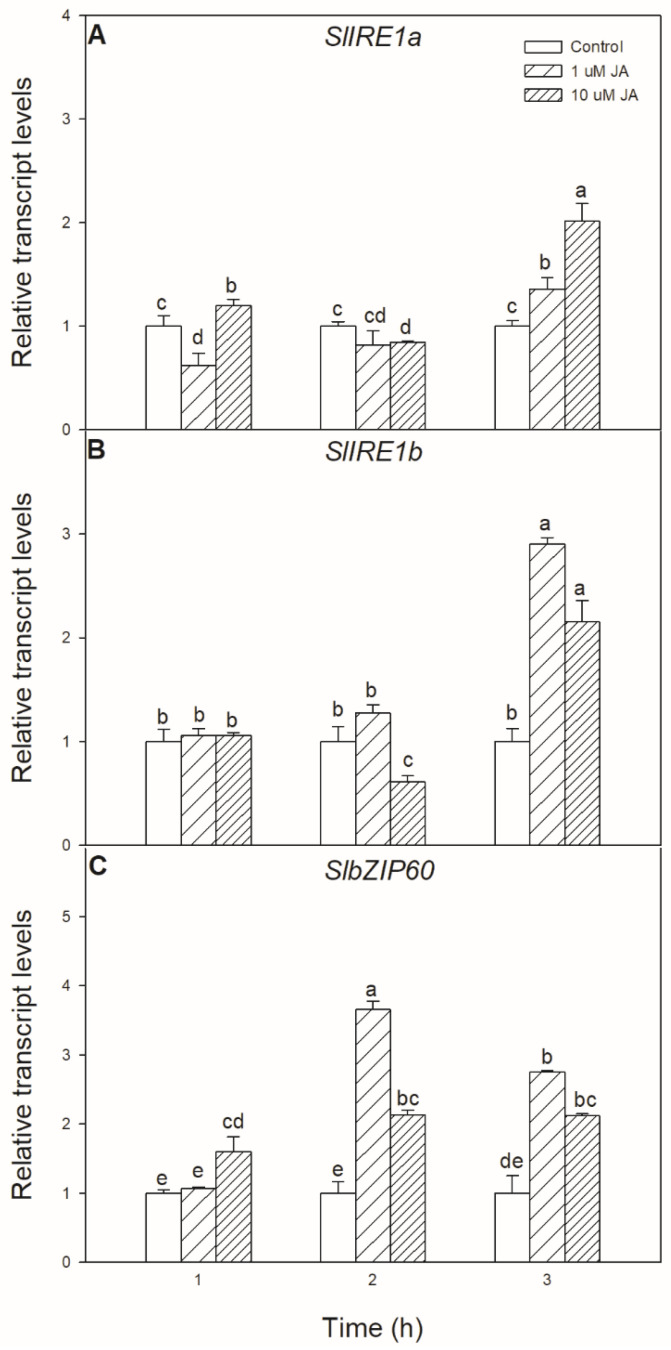
Changes in the transcript accumulation of *SlIRE1a* (**A**), *SlIRE1b* (**B**), and *SlbZIP60* (**C**) in the leaves of wild-type tomato plants exposed to 1 or 10 µM jasmonic acid (JA) for 6 h. Results are the means ± SE. Data with different letters indicate significant differences at *p* < 0.05 level, *n* = 3.

Exogenous JA treatment has not only a significant effect on UPR but also could modulate the transcript levels of ER stress signalling components. Significant changes were found in the transcript accumulation of *SlIRE1a* and *SlIRE1b*, as well as in the transcript levels of *SlbZIP60* upon exogenous JA treatment, which were all elevated after 6 h in the leaves of tomato plants (Figure 2).

**Figure 3:**
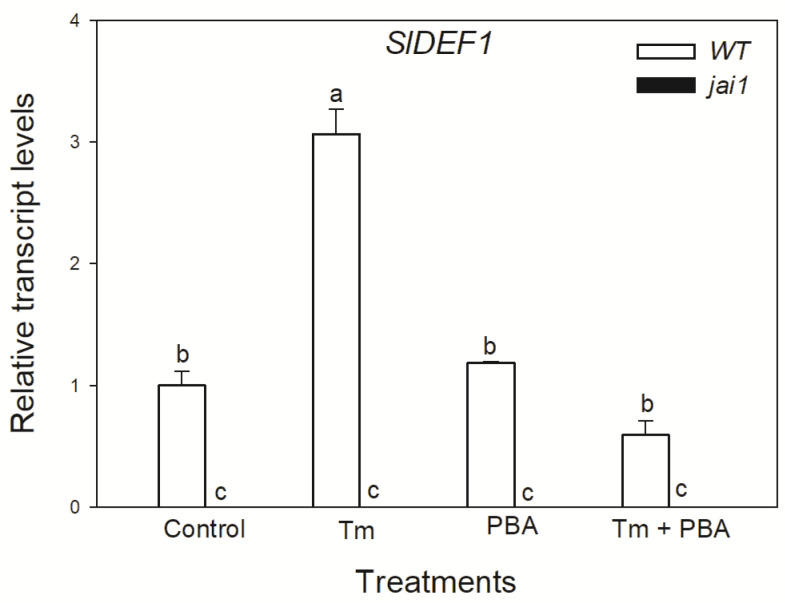
Changes in the transcript accumulation of *SlDEF1* in the leaves of wild-type (WT) and JA signalling mutant (jai1) tomato plants exposed to 5 µg mL^−1^ tunicamycin (Tm), 1 mM 4-phenylbutyrate (PBA) and Tm + PBA for 6 h. Results are the means ± SE. Data with different letters indicate significant differences at *p* < 0.05 level, *n* = 3.

Based on these observations, we were interested in whether JA might play a role in ER stress and UPR in tomato leaves. To detect the effects of JA in these processes, wild-type (WT) and JA signalling mutant (jai1) tomato plants were used. Firstly, changes in the transcript levels of JA marker gene defensin (*SlDEF1*) were detected. Treatment of the ER stress inducer Tm significantly elevated the amount of transcript in WT leaves (Figure 3). At the same time, the application of chemical chaperon PBA significantly reduced this increment in *SlDEF1* transcript levels. The transcription of *SlDEF1* in *jai1* leaves was not detected (Figure 3).

**Figure 4:**
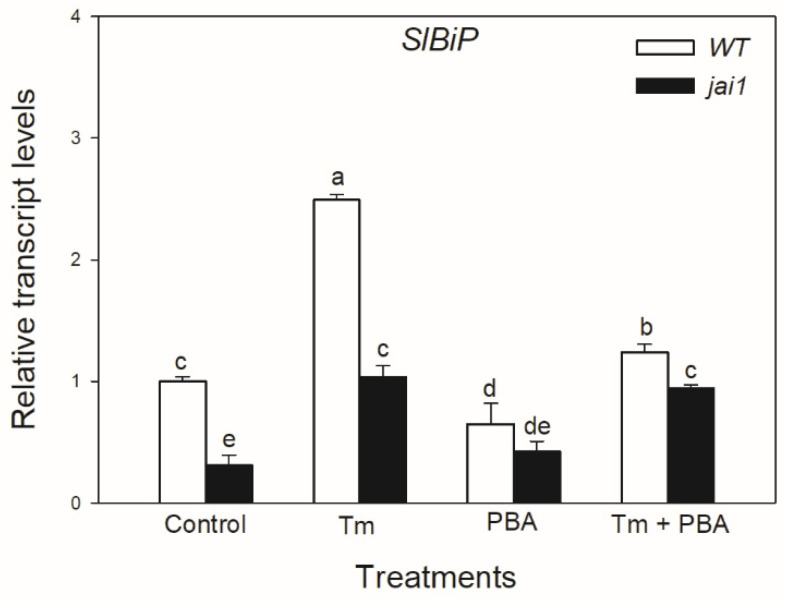
Changes in the transcript accumulation of *SlBiP* in the leaves of wild-type (WT) and JA signalling mutant (jai1) tomato plants exposed to 5 µg mL^−1^ tunicamycin (Tm), 1 mM 4-phenylbutyrate (PBA), and Tm + PBA for 6 h. Results are the mean ± SE. Data with different letters indicate significant differences at *p* < 0.05 level, *n* = 3.

Quantitative RT-PCR analysis indicated that the transcript accumulation of *SlBiP* was significantly induced by Tm treatment in WT plants, whereas interestingly, this was significantly lower in *jai1* leaves (Figure 4). The accumulation of *SlBiP* transcripts was suppressed by the use of chemical chaperone PBA in both tomato genotypes, suggesting that Tm treatment was a major cause of the induction of UPR (Figure 4).

**Figure 5:**
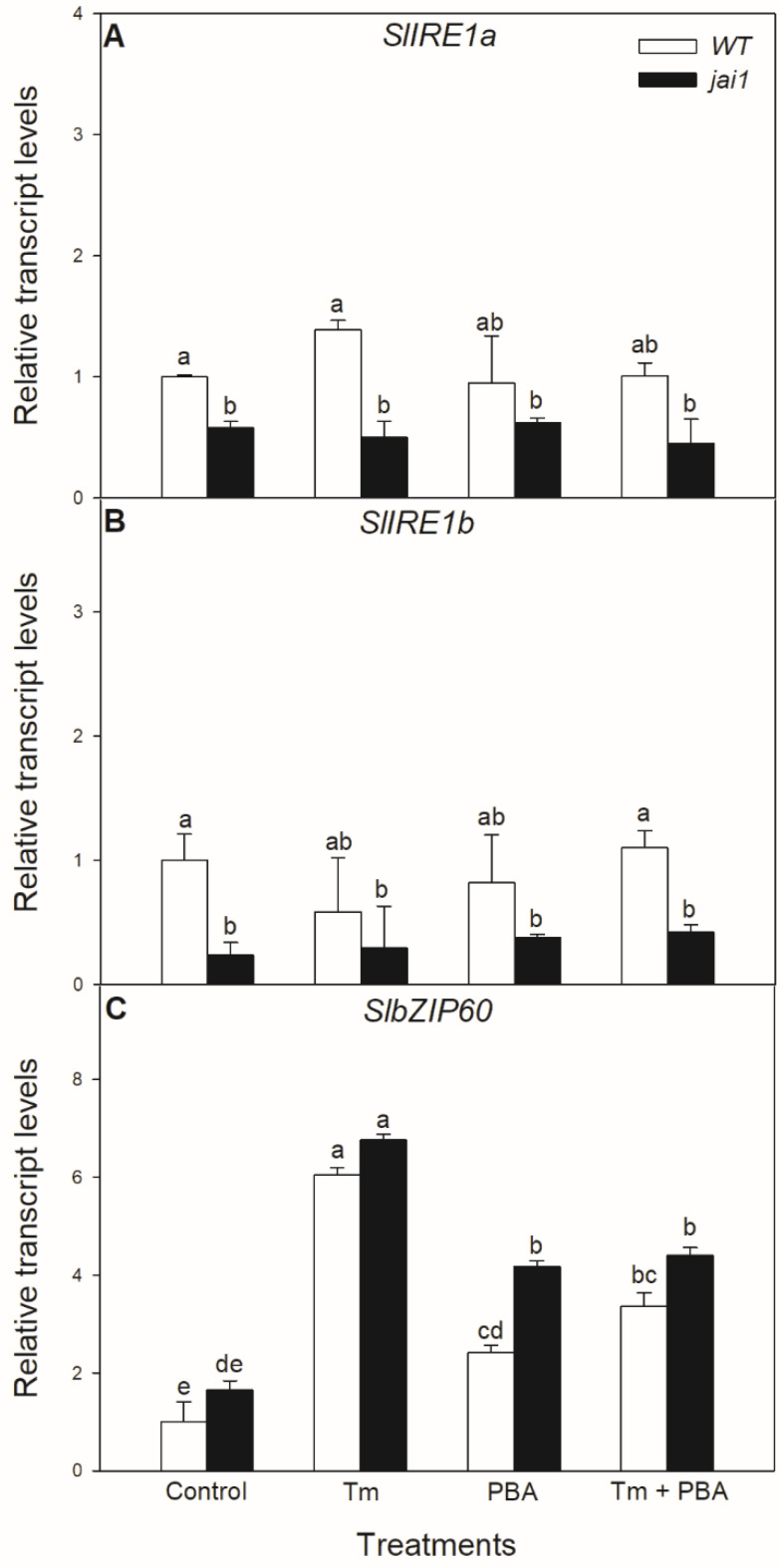
Changes in the transcript accumulation of *SlIRE1a* (**A**), *SlIRE1b* (**B**) and *SlbZIP60* (**C**) in the leaves of wild-type (WT) and JA signalling mutant (jai1) tomato plants exposed to 5 µg mL^−1^ tunicamycin (Tm), 1 mM 4-phenylbutyrate (PBA), and Tm + PBA for 6 h. Results are the means ± SE. Data with different letters indicate significant differences at *p* < 0.05 level, *n* = 3.

Similar effects of these treatments were not observed in the relative transcript accumulation of the coding sequences of ER stress mediator IRE1. The transcript levels of *SlIRE1a* and *SlIRE1b* did not change significantly after the 6-h-long Tm treatment in the investigated tomato genotypes but transcript levels of *SlIRE1a* and *SlIRE1b* genes were basically lower in *jai1* leaves (Figure 5). In contrast to the transcript levels of *SlIRE1a* and *SlIRE1b*, the relative transcript levels of *SlbZIP60* changed similarly to *SlBiP* in WT leaves, but it was basically higher in *jai1* compared to WT plants. The accumulation of transcript of *SlbZIP60* increased by every treatment in *jai1* leaves (Figure 5).

**Figure 6:**
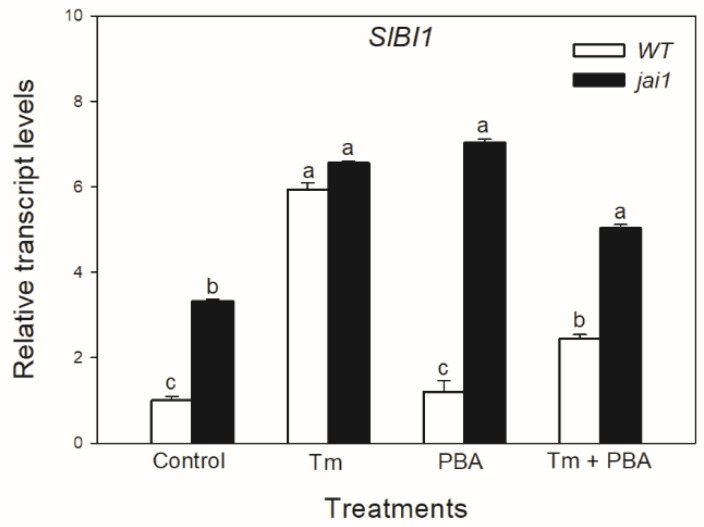
Changes in the transcript accumulation of *Bax Inhibitor-1* (*SlBI1*) in the leaves of wild-type (WT) and JA signalling mutant (jai1) tomato plants exposed to 5 µg mL^−1^ tunicamycin (Tm), 1 mM 4-phenylbutyrate (PBA) and Tm + PBA for 6 h. Results are the means ± SE. Data with different letters indicate significant differences at *p* < 0.05 level, *n* = 3.

In contrast to the changes in *SlIRE1a* and *SlIRE1b* transcripts, the relative transcript levels of *SlBI1* changed similarly to *SlBiP* and *SlbZIP60* in WT leaves, Tm increased the accumulation of *SlBI1* transcripts, but the combined treatment with PBA decreased it (Figure 5). Interestingly, transcript levels of *SlBI1* were basically higher in *jai1* compared to WT leaves similarly to *SlbZIP60*, which were elevated by every treatment (Figure 6).

**Figure 7:**
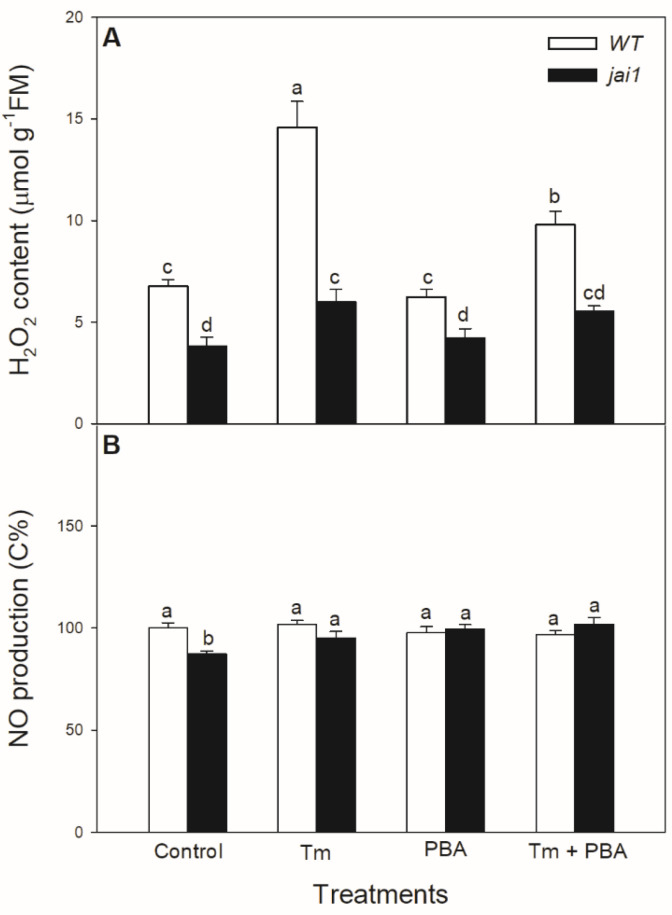
Changes in H_2_O_2_ content (**A**) and NO production (**B**) in the leaves of wild-type (WT) and JA signalling mutant (jai1) tomato plants exposed to 5 µg mL^−1^ tunicamycin (Tm), 1 mM 4-phenylbutyrate (PBA), and Tm + PBA for 6 h. Results are the means ± SE. Data with different letters indicate significant differences at *p* < 0.05 level, *n* = 3.

The potential JA-dependent signalling components were also analysed in the ER-stress response. Significant increase in the H_2_O_2_ level was measured in the Tm-treated WT plants, while it exhibited much moderate increase upon Tm treatment in *jai1* leaves (Figure 7A). Application of the combined treatment of Tm + PBA significantly decreased this increment in H_2_O_2_ content (Figure 7A). Interestingly, H_2_O_2_ levels were basically lower in *jai1* compared to WT leaves, similarly to NO production. At the same time, the production of NO changed neither by Tm nor by PBA in the investigated genotypes at this time point (Figure 7B).

**Figure 8:**
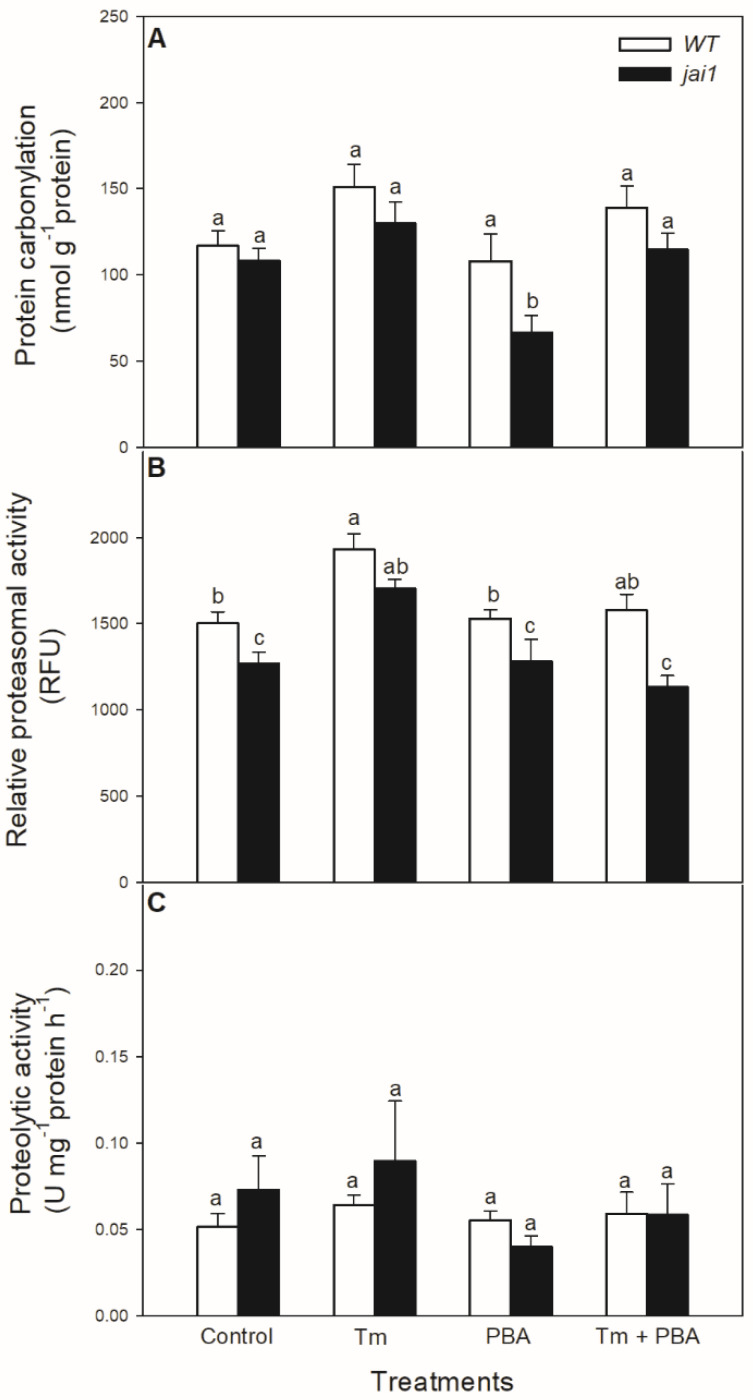
Changes in protein carbonylation (**A**), proteasomal activity (**B**), and total proteolytic activity (**C**) in the leaves of wild-type (WT) and JA signalling mutant (jai1) tomato plants exposed to 5 µg mL^−1^ tunicamycin (Tm), 1 mM 4-phenylbutyrate (PBA), and Tm + PBA for 6 h. Results are the means ± SE. Data with different letters indicate significant differences at *p* < 0.05 level, *n* = 3.

The potential effects of oxidative stress generated by Tm on proteins were also investigated. Protein carbonylation did not change significantly after the 6-h-long Tm treatment (Figure 8A), but the chemical chaperone PBA decreased it in *jai1* leaves. In contrast to these findings, Tm increased the relative proteasomal activity in both genotypes, which was significantly declined by the co-application with PBA (Figure 8B). However, the relative proteasomal activity was significantly lower in *jai1* leaves (Figure 8B). At the same time, the total proteolytic activity did not change significantly after 6 h upon the treatments (Figure 8C).

**Figure 9:**
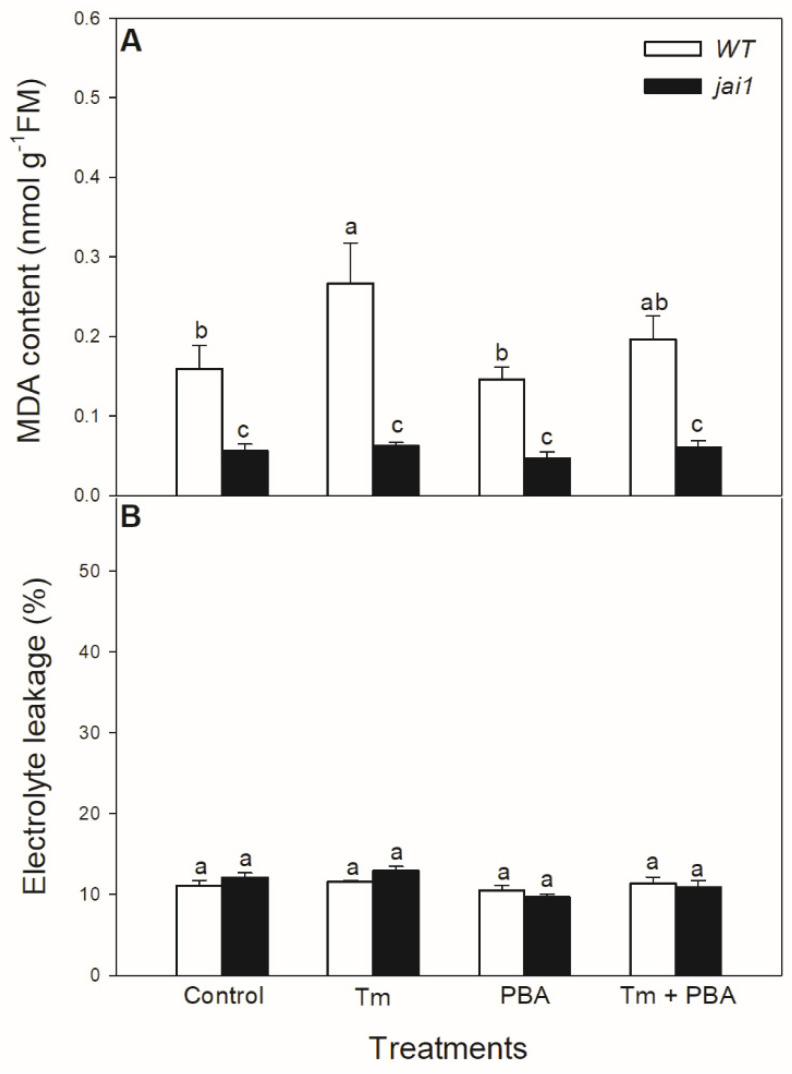
Changes in malondialdehyde (MDA) content (**A**) and electrolyte leakage (**B**) from the leaves of wild-type (WT) and JA signalling mutant (jai1) tomato plants exposed to 5 µg mL^−1^ tunicamycin (Tm), 1 mM 4-phenylbutyrate (PBA), and Tm + PBA for 6 h. Results are the means ± SE. Data with different letters indicate significant differences at *p* < 0.05 level, *n* = 3.

Lipid peroxidation was measured based on the changes in MDA content. Tm significantly elevated MDA content in WT leaves, but PBA treatment reduced this change (Figure 9A). Interestingly, MDA content did not alter after the treatments of ER stress modulators in *jai1* leaves (Figure 9A). Cell death induced by Tm was estimated by the measurement of electrolyte leakage, which did not show any significant differences upon the treatments after 6 h in the investigated genotypes (Figure 9B).

## 4. Discussion

Despite the fact that the molecular mechanism of ER stress and UPR has been intensively studied in the past years, there are many gaps in comprehending the interaction of ER stress signalling and response mediated by several phytohormones such as JA. It is well-known that JA regulates many processes in plant cells such as defence hormone biosynthesis, nucleic acid metabolism, lipid metabolism, and sugar metabolism, photosynthesis, senescence, and the activation of transporters or kinases [60]. At the same time, there are many results in transcriptome analysis which confirmed that JA plays a crucial role in ROS metabolism, proteasome, and protease activities, as well as chaperon synthesis in various plant species [60,61,62,63,64], suggesting the key role of JA in UPR. In this study, we investigated for the first time the potential role and effects of JA in ER stress and UPR in the cultivated, intact tomato plants based on the analysis of transcript levels of selected marker genes using qRT-PCR [30,33]. The physiological effects of the application of ER stress-inducing agent Tm and changes in JA-dependent signalling components are also the focus of our investigations.

JA plays critical role in protecting plants from various pathogens or insects and in limiting damage from several abiotic stressors [34,35,36,37]. Rapid changes in JA levels in the infected or damaged organs could be crucial for the plants to survive [35,38,39]. Several authors proved that JA accumulation and JA-mediated gene expression occur within several hours after the pathogen infection [65,66,67,68]. The results of Pozo et al. [69] showed the transcript levels of the JA marker *Lipoxygenase2* (*LOX2*) started to rise at 1 h after exogenous 50 mM MeJA treatment and reached the maximum at 6 h, but it significantly decreased after 12 h. Based on these observations, the effects of JA at the 6^th^ hour were selected to investigate the physiological role of JA under ER stress in more detail. Moreover, this time is enough to effectively induce ER stress and UPR in plants e.g., in the case of SA [43,70,71]. JA and its derivatives induce the production of a wide range of antifungal proteins such as defensins or protease inhibitors, as well as activate defence enzymes related to phytoalexin synthesis, such as chalcone synthase, phenylalanine ammonia-lyase, and hydroxymethylglutaryl-coenzyme A reductase [37,40]. Fast and optimal coordination of the expression or the regulation of protein secretory machinery is required to ensure the folding, modification, and transport of proteins, which are mediated by JA similarly to SA [4,29,70]. UPR is dependent on molecular chaperones such as BiPs, which are the key components responsible for protein folding, assembly, translocation, and degradation under normal and stress conditions [72,73]. Our results showed that the micromolar concentration of exogenous JA significantly induced the transcript accumulation of *BiP* within 6 h in the leaves of tomato plants. This could have great importance because earlier, it has been found only that the exogenous application of JA or SA in millimolar concentration increased the expression of BiP genes in *Arabidopsis* [33] or in soybean [30]. In this work, JA was applied in two different micromolar concentrations, which could be closer to the natural endogenous changes in plants upon pathogen infection [74,75]. Moreover, it can be described the first time that the effects of different concentrations of JA showed time-dependent changes on *SlBiP* transcription, which can be related to the concentration-dependent transport and signalling of JA similarly to SA [32,76]. Similar to this, it has been also observed that the transcript levels of *BiP* were varied and not linearly elevated by TM as a function of time [77]. In addition, both exogenous JA treatments induced the transcript accumulation of the coding sequences of ER stress sensor IRE1 and UPR-activating transcription factor bZIP60 in tomato, suggesting that JA not only plays a role in UPR, but it has also a significant effect on ER stress signalling components. Unfortunately, only IRE1 and bZIP60 have been identified clearly in the tomato genome among the ER stress signalling components described in *Arabidopsis* [49], and the precise identification of other components (e.g., bZIPs, NACs) in this process is required to understand the complex molecular mechanism of ER stress sensing and signalling in tomato.

Our results provide evidence for the first time that exogenous JA can generate ER stress and UPR in tomato. The potential role of JA in these processes was further analysed by WT and JA signalling mutant (*jai1*) tomato plants. To test the potential role of JA in UPR, the exogenous application of several chemicals such as Tm, the inhibitor of N-glycosylation of secreted glycoproteins, and the chemical chaperon PBA were used. These modulators have been applied extensively under laboratory conditions [14]. Earlier, it was found that the application of Tm induced ER stress within 2 h in *Arabidopsis* leaves [42], and 1 mM PBA was able to significantly reduce it [47]. Our results demonstrate that Tm induced the accumulation of JA marker gene defensin (*SlDEF1*) in WT leaves, which was inhibited by the application of PBA. These changes in transcripts of *SlDEF1* could confirm that Tm induced JA signalling in the leaves of WT tomato plants. In addition, Tm also significantly induced the transcript accumulation of *SlBiP* in WT plants, which was significantly lower in *jai1* leaves, suggesting a significant role of JA in UPR. The accumulation of *SlBiP* transcript was suppressed by PBA in both tomato genotypes, indicating that Tm treatment was a major cause of the induction of UPR. The close relationship between BiP chaperone, JA, and other defence hormones was confirmed recently in BiP-overexpressing transgenic soybean plants, where the JA content was lower compared to the WT under control conditions [41]. Others also found that JA plays a role in the induction of various chaperon-coding sequences in different plant species [60,61]. While the transcript levels of *SlIRE1a* and *SlIRE1b* did not change significantly at this time point after Tm treatment in none of the tomato genotypes, the transcript levels of both ER stress sensor genes were basically lower in *jai1* leaves, suggesting a principal role of JA in the regulation of IRE. At the same time, the insignificant changes in the transcript accumulation of *IRE1* did not show the JA-mediated activity of IRE1 after Tm treatment in WT. Moreover, the transcript accumulation of these genes could be also dependent on the time similarly to the results measured in the case of exogenous JA treatments. It can be concluded that describing the direct effects of JA on the molecular mechanism of ER stress sensing and signalling needs further research in tomato. In contrast to the results of *SlIRE1* transcription, the transcript accumulation of *SlbZIP60* changed similarly to *SlBiP*, but transcript levels of *SlbZIP60* were higher in *jai1* leaves. These results showed that Tm induced the transcript accumulation of *SlbZIP60*, but this was independent on JA based on the measurements in *jai1*. Two copies of IRE1, IRE1a and IRE1b, can be found in *Arabidopsis* with diverse function under ER stress. IRE1a is required for bZIP60 splicing upon pathogen infection, while IRE1b plays a role in bZIP60 processing upon Tm-induced ER stress [42]. In tomato, further studies are needed to explore the adequate role of the two IRE1 sequences and IRE1-mediated bZIP60 pathway under ER stress and UPR. Moreover, further research studies are required to identify other signalling components of ER stress and UPR in tomato, which could be different compared to *Arabidopsis*.

Surprisingly, in contrast to *SlIRE1a* and *SlIRE1b* but similarly to *SlbZIP60,* the relative transcript levels of *SlBI1* were basically higher in *jai1* compared to WT leaves. Treatment with Tm increased the transcript accumulation of *SlBI1* but the combined treatment with PBA decreased it; these changes were more pronounced in *jai1* leaves, suggesting the inhibitory effects of JA on *SlBI1* under ER stress. It is known that BI1 is involved in the inhibition of PCD in *Arabidopsis* by decreasing the ER stress-induced ROS production or by regulating Ca^2+^ homeostasis [47,78]. Moreover, BI1 can increase resistance to necrotrophic pathogens controlled by JA [79]. Elevated JA content and the up-regulation of JA- and SA-related genes at the initial and/or late stages of the infection may suppress the anti-cell death mechanism. In contrast to this, the exogenous application of SA induced the transcript accumulation of *SlBI1* in a concentration-dependent manner [80]. It was also found that over-expression of the *AtBI1* gene delayed the onset of MeJA-induced leaf senescence in *Arabidopsis*, suggesting a complex relationship between BI1 and JA [81].

The potential role of JA-dependent signalling components such as H_2_O_2_ and NO was also analysed in the ER stress response. It has been shown earlier that there is a tight association between ROS and ER stress [7,82]. H_2_O_2_ is the most stable ROS, which can act both as a toxic compound (inducing oxidative stress and cell death by oxidizing proteins and increasing lipid peroxidation) or as a signalling molecule (mediating tolerance by inducing antioxidant enzymes) in a concentration-dependent manner [83,84,85]. There is also a close interaction between ROS/NO signalling and JA [40,86,87]. It has been shown that elicitor treatment or wounding trigger ROS production through NADPH oxidase activation can induce the expression of JA biosynthesis genes [88,89]. In addition, ROS accumulation is required for JA-induced stomatal closure in *Arabidopsis* [90,91]. We also measured a significant increase in H_2_O_2_ after the treatment with Tm similarly to another research group [43], but we observed for the first time that H_2_O_2_ levels stayed significantly lower in *jai1* compared to WT leaves, suggesting the role of JA in controlling ER stress sensing and signalling. The application of PBA together with Tm was able to decrease this increment in H_2_O_2_ content. Interestingly, H_2_O_2_ levels were basically lower in *jai1* compared to WT leaves. Similarly to our result, lower H_2_O_2_ content was measured in defenseless-1 (def-1) JA-deficient tomato mutant compared to WT plants [92]. NO is also an important signal molecule involved in multiple plant physiological processes [93,94]. It was earlier found that NO contributes positively to elicit the production of jasmonates [95] and JA enhances NO synthesis in guard cells [91]. At the same time, NO production did not change after treatments of Tm and PBA in intact leaves in either of the tomato genotypes, suggesting that it is not a component of ER stress signalling and UPR at this time point after the treatments in tomato leaves.

High concentrations of ROS can induce the accumulation of oxidized proteins [83]. To avoid their toxic accumulation, carbonylated proteins are degraded through the action of the 26S proteasome in the cytosol [96]. It was earlier described that Tm significantly elevated H_2_O_2_ levels and increased protein carbonylation but only after 48 h in *Arabidopsis* [43]. Based on our observation, protein carbonylation did not change significantly after the 6-h-long Tm treatment but elevated slightly in WT leaves. Interestingly, PBA treatment significantly decreased it in *jai1* leaves. In contrast to protein carbonylation, the ER stress generator Tm elevated the relative proteasomal activity in both tomato genotypes, which was significantly suppressed by the addition of PBA. At the same time, proteasomal activity was significantly lower in *jai1* leaves, suggesting a role of JA in the coordinated degradation of oxidized proteins. However, total proteolytic activity, which is elevated under cell death induction by ER stress or immune response [10,97,98], did not change after the 6-h-long treatment of ER stress modulators in intact leaves of tomato plants. Based on the results, it can be concluded that only the regulated proteolytic activity was initiated, and cell death was not promoted at this time point.

At the same time, lipid peroxidation is elevated by Tm treatment in the leaves of tomato, as it was shown in the measurement of MDA content. The application of PBA could reduce this increment in MDA level upon Tm treatment. This is in good accordance with the earlier result in *Arabidopsis*, where significantly higher lipid peroxidation was also measured, following the Tm treatment [43]. Interestingly, MDA content did not change after the treatments of ER stress modulators in *jai1* leaves, suggesting the role of JA in ER stress-induced oxidative burst sensing and signalling. Similarly to our results, JA-deficient defenseless-1 (def-1) mutant tomato showed basically lower MDA content compared to WT plants [92]. In contrast to the elevated MDA content, cell membranes remained intact upon Tm treatments and cell death was not initiated during the 6 h-long Tm treatment in the leaves of neither tomato genotypes based on the results of the electrolyte leakage measurements.

## 5. Conclusions

Our results confirmed the first time that JA plays a role in ER stress sensing and signalling in intact tomato plants. Exogenous JA not only induces the transcript accumulation of UPR marker gene *SlBiP* but also elevates the transcript levels of ER stress sensor *SlIRE1* and the UPR-related basic transcription factor *SlbZIP60* after 6 h, but these changes were concentration- and time-dependent, respectively. It was the first time when the role of JA in Tm-induced ER stress and UPR was investigated in *jai1* tomato plants, which are defective in JA signalling. Tm induced significant transcript accumulation in *SlBiP*, *SlbZIP60,* and *SlBI1*, which was dependent on JA based on the measurements in *jai1* leaves. We found at the first time that H_2_O_2_ content, proteasomal activity, and lipid peroxidation induced by Tm is regulated by JA, while NO seems to be uninvolved in ER stress and UPR signalling at this time point in tomato leaves. Our results suggested the first time the significant physiological effects of JA in ER stress and UPR in tomato leaves, but the concrete molecular mechanism of JA in ER stress and UPR needs further research.

## Figures and Tables

**Table 1:** Primer pairs used for qRT-PCR.

Gene (Locus)	Reverse/Forward	Sequence (5′-3′)
*SlBiP* (L08830)	Reverse	5′-TCAGAAAGACAATGGGACCTG-3′
	Forward	5′-GCTTCCACCAACAAGAACAAT-3′
*SlDEF1* (NM_001346524)	Reverse	5′-GGCACAATCCATTCGTTTCT-3′
	Forward	5′-TTGGTCCCATTTCAGTAGCC-3′
*SlIRE1a* (XM_004232529)	Reverse	5′-CGCTTTACATCACCAGGACA-3′
	Forward	5′-CAGCAGCCCCTATTTCAGC-3′
*SlIRE1b* (XM_004238397)	Reverse	5′-CCAGTCCTCTATTGCCTCTCA-3′
	Forward	5′-CCTGACCTAAATGCCCAGAT-3′
*SlbZIP60* (XM_004238421.3)	Reverse	5′-TTGCTGCCGAATCTCTTTCT-3′
	Forward	5′-CGACTGGGAAACCTTGTGTT-3′
*SlBI1* (NM_001247521)	Reverse	5′-CTCAAAAACTTCCGCCAGAT-3′
	Forward	5′-GCCACTAAAGCACAGCATAGC-3′
